# Effect of behavioural practice targeted at the motor action selection network after stroke

**DOI:** 10.1111/ejn.15754

**Published:** 2022-07-17

**Authors:** Jill Campbell Stewart, Jessica F. Baird, Allison F. Lewis, Stacy L. Fritz, Julius Fridriksson

**Affiliations:** ^1^ Department of Exercise Science University of South Carolina Columbia South Carolina USA; ^2^ Department of Communication Sciences and Disorders University of South Carolina Columbia South Carolina USA

**Keywords:** action selection, brain imaging, motor planning, stroke, upper extremity

## Abstract

Motor action selection engages a network of frontal and parietal brain regions. After stroke, individuals activate a similar network, however, activation is higher, especially in the contralesional hemisphere. The current study examined the effect of practice on action selection performance and brain activation after stroke. Sixteen individuals with chronic stroke (Upper Extremity Fugl–Meyer motor score range: 18–61) moved a joystick with the more‐impaired hand in two conditions: Select (externally cued choice; move right or left based on an abstract rule) and Execute (simple response; move same direction every trial). On Day 1, reaction time (RT) was longer in Select compared to Execute, which corresponded to increased activation primarily in regions in the contralesional action selection network including dorsal premotor, supplementary motor, anterior cingulate and parietal cortices. After 4 days of practice, behavioural performance improved (decreased RT), and only contralesional parietal cortex significantly increased during Select. Higher brain activation on Day 1 in the bilateral action selection network, dorsolateral prefrontal cortex and contralesional sensory cortex predicted better performance on Day 4. Overall, practice led to improved action selection performance and reduced brain activation. Systematic changes in practice conditions may allow the targeting of specific components of the motor network during rehabilitation after stroke.

## Graphical abstract

After 4 days of practice, individuals post‐stroke improved action selection behavioural performance with a corresponding decrease in brain activation throughout the action selection network, especially in the contralesional hemisphere. Higher brain activation at baseline in the bilateral action selection network as well as prefrontal and sensory cortices predicted better performance on Day 4. Systematic changes in practice conditions may allow the targeting of specific components of the motor network during rehabilitation after stroke.

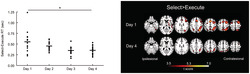

**Abbreviations**
ACC: anterior cingulate cortex
CST: corticospinal tract
FA: fractional anisotropy
LBD: left brain damage
MRI: magnetic resonance imaging
PMd: dorsal premotor
RBD: right brain damage
RT: reaction time
SMA: supplementary motor area
TE: echo time
TR: repetition time
UE FM: upper extremity Fugl–Meyer

## 1 INTRODUCTION

Elevated brain activation during movement after stroke has been well described. Overall, brain activation tends to be higher in individuals post‐stroke compared to age‐matched individuals without stroke, even for the performance of simple motor tasks (Buma et al., 2010; Carey et al., 2002; Cramer et al., 1997; Grefkes & Ward, 2014; Rehme et al., 2012; Ward et al., 2003). When motor task demands are increased in nondisabled individuals, brain activation increases (Catalan et al., 1998; Cramer et al., 2002; Verstynen et al., 2005; Winstein et al., 1997), including in the hemisphere ipsilateral to movement (Barany et al., 2020; Buetefisch et al., 2014). The increase in activation in response to increased task demands is even greater after stroke with individuals often engaging both the ipslesional and contralesional hemispheres (Dennis et al., 2011; Schaechter & Perdue, 2008). This pattern of increased activation to meet task demands after stroke may or may not benefit task performance. However, the effect of behavioural practice on this activation pattern in response to more complex task conditions after stroke has not been reported.

Skilled performance of motor tasks involves a variety of movement preparation processes that engage a range of brain regions (Andersen & Cui, 2009; Cisek & Kalaska, 2010; Scott, 2012). Action selection, or selection of a motor response, is one important movement preparation process (Cisek & Kalaska, 2010). When action selection demands are added to movement through abstract, visual cues, a network of brain regions are activated including premotor, frontal and parietal cortices, with dorsal premotor cortex (PMd) shown to be a key node in this network (Grafton et al., 1998; Grol et al., 2006; Halsband & Passingham, 1985; O'Shea et al., 2007; Toni et al., 2002). After stroke, a similar network is activated during a motor action selection task, but regions in the contralesional hemisphere ipsilateral to the moving hand are primarily engaged to meet the action selection demands (Stewart et al., 2016). This bilateral activation pattern is similar to that of other movement tasks after stroke (Rehme et al., 2012). However, the effect of practice on activation during action selection and whether baseline brain activation predicts response to a period of motor action selection practice is not known. Additionally, side of brain damage can impact the deficits in movement preparation seen after stroke (Haaland & Harrington, 1989; Schaefer et al., 2009; Stewart, Gordon, & Winstein, 2014; Tretriluxana et al., 2009). Previous studies have suggested that the left hemisphere has a dominant role in action selection in right hand dominant individuals (Cavina‐Pratesi et al., 2006; Rushworth et al., 1998; Schluter et al., 1998). However, the effect of side of brain damage on action selection performance with the more impaired arm and changes with practice are not fully known.

Behavioural practice is the cornerstone of rehabilitation after stroke. While higher doses or repetitions of practice may or may not benefit motor recovery (Lang et al., 2016; Winstein et al., 2019), the optimal content, focus and intensity of these repetitions remain unknown. Systematic changes in movement task conditions lead to changes in neural activation that are condition‐specific after stroke (Askim et al., 2010; Dennis et al., 2011; Dodakian et al., 2014; Schaechter & Perdue, 2008), however, the effect of practice on this activation has not been systematically investigated. Behavioural practice conditions that target a specific motor preparation process and its related neural network, such as through the addition of action selection demands, may provide an avenue to challenge a specific component of the motor network during training and improve outcomes (Cramer et al., 2011; Dodakian et al., 2013).

The purpose of this study was to determine the effect of motor practice targeted at the action selection network on behavioral performance and neural activation after stroke. We hypothesized that action selection performance would improve after a period of practice as shown by improved behavioral performance and decreased activation in the action selection network, especially in the contralesional hemisphere. We also hypothesized that initial activation in the ipsilesional action selection network would predict performance at the end of practice. Specifically, we expected that individuals who showed higher activation in the ipsilesional action selection network at baseline, suggesting greater ability to engage the ipsilesional network early in practice, would have better action selection behavioral performance at the end of practice. Finally, we explored differences in action selection performance and changes with practice between individuals with left hemisphere versus right hemisphere stroke.

## 2 MATERIALS AND METHODS

Individuals with motor deficits due to stroke practiced a motor action selection task on four consecutive days. On Days 1 and 4, participants completed the action selection task during functional magnetic resonance imaging (MRI) to examine the neural correlates of task performance. Clinical measures of arm and hand function were completed on a separate day prior to the first day of motor practice.

### 2.1 Participants

Sixteen individuals in the chronic stage of stroke were recruited from the local community. We aimed to recruit individuals with residual motor deficits but with some degree of hand grasp ability to allow completion of the experimental task. Individuals were eligible to participate if they were ≥18 years old, had a stroke at least 6 months prior to enrollment, were right‐hand dominant (Oldfield, 1971) prior to stroke, showed evidence of upper extremity impairment as defined by an upper extremity Fugl‐Meyer (UE FM) score (Fugl‐Meyer et al., 1975) <66 and/or at least a 15% deficit on the Nine Hole Peg Test (Mathiowetz, Weber, et al., 1985) on the more impaired hand compared to the less impaired hand, demonstrated some movement ability as shown by an UE FM score >30 and/or the ability to move at least one block on the Box and Blocks Test (Mathiowetz, Volland, et al., 1985) and scored ≥19 on the Montreal Cognitive Assessment (Nasreddine et al., 2005) in individuals without expressive aphasia. Individuals were excluded if they had any acute medical problems, severe ideomotor apraxia as defined by a score ≤65 on the Test of Upper Limb Apraxia (Vanbellingen et al., 2010), hemispatial neglect with a score <52 on the Behavioral Inattention Test Star Cancellation (Hartman‐Maeir & Katz, 1995), significant arm pain that interfered with movement, contraindications to MRI scanning (e.g., metal implants or claustrophobia), or a history of other, non‐stroke related neurological disorder. All participants provided written‐informed consent on a form approved by the University of South Carolina Institutional Review Board.

### 2.2 Motor action selection task

**FIGURE 1:**
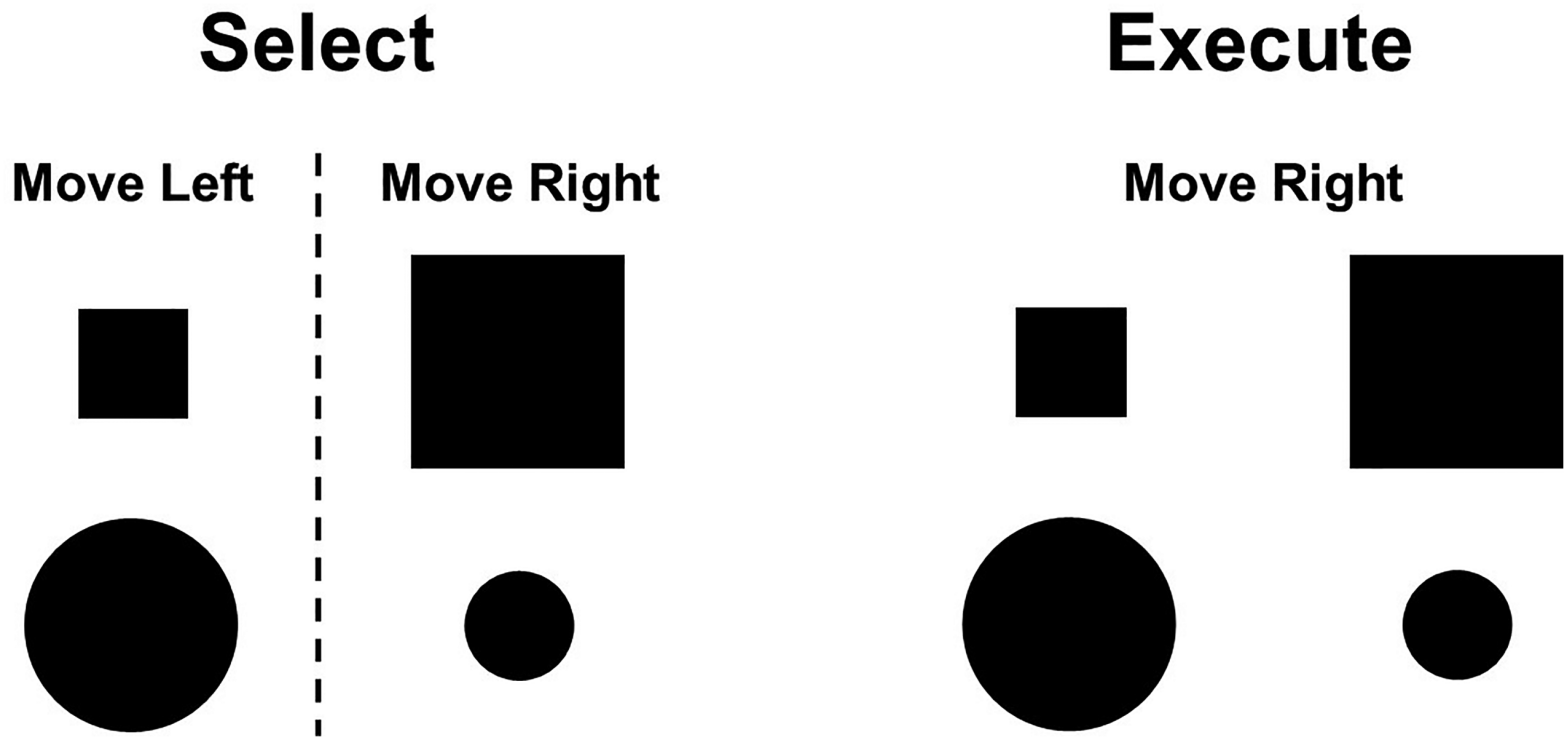
Select and Execute cues provided during the motor action selection task. During Select, movement direction was dictated by an abstract rule (large square or small circle = move right; small square or large circle = move left; the large square/circle was twice the size of the small square/circle). During Execute, movement direction was the same on every trial regardless of visual cue. Movement direction for the Execute condition (right or left) was counterbalanced across participants. During functional MRI, the cues were the same, but the cues were green during the 24 s movement blocks and red during the 24 s rest blocks.

All participants performed the motor action selection task with the more impaired hand. The task involved movement of a standard joystick (maximum excursion 20°; spring that returned position to center) based on a visual cue presented on a laptop screen using E‐Prime 2.0 (Psychology Software Tools, Inc., Sharpsburg, PA) in two different conditions: Select and Execute. In the Select condition, the individual moved right or left based on an abstract rule (externally cued choice response task). When a small square or large circle was shown, a joystick movement to the left was made; when a large square or small circle was shown, a joystick movement to the right was made (Figure 1). In the Execute condition, the visual cues were the same; however, the individual made a joystick movement in the same direction on every trial irrespective of the size/shape of the cue (no rule to follow, simple response task). Movement direction for the Execute condition was counterbalanced across participants. In both conditions, a single cue was presented for 2 s in a random order; the inter‐trial interval varied between 2.0 and 3.5 s to minimize anticipatory responses prior to the cue. Only the cue that directed the movement response was presented on the screen; there was no target to capture and therefore no movement spatial–temporal demands beyond the direction of movement (right/left). No feedback was provided during practice, however, directions (i.e., the rule for selection of the movement response) were provided at the start of every practice block. The joystick was positioned directly next to the laptop, and participants were allowed to grasp the joystick in whatever way they chose. Additional support was provided when needed to help keep the hand on the joystick (e.g., support under the elbow).

On Day 1, participants completed a practice session of both task conditions prior to the MRI. Verbal and visual instruction on the Select condition was provided followed by a practice block (24 trials) of the Select condition. Three blocks of 24 trials each were then completed in each condition in alternating order; the condition completed in the first block (Select or Execute) was counterbalanced across participants. On subsequent, consecutive days (Days 2 through 4), participants practiced the motor task in the laboratory for 10 blocks per day (5 blocks in each condition in alternating order) for a total of 96 trials per condition on each day. Data collected in the laboratory were used to quantify task performance across days.

After completion of the training blocks on Days 1 and 4, the participant practiced the MRI version of the task prior to the MRI. This version alternated periods of movement (cues were green) with periods of rest (cues were red) in a block design (see below) and included a total of 10 movement trials per run. During scanning, some participants held an MRI compatible joystick but others did not due to space limitations (joystick would not fit) or an inability to maintain a grip on the device (*N* = 3; Subject IDs 5, 6 and 16); these individuals held a simple foam cylinder that was not attached to a base in the same position as the joystick and moved the top of the cylinder to the right or left as indicated by the cue. During all runs, a researcher observed hand movement and documented responses (right or left movement) to determine the accuracy of the movement direction.

### 2.3 Brain image acquisition

On Days 1 and 4, individuals completed the motor action selection task in a 3T Siemans Prisma MRI scanner with a 20‐channel head coil. Functional MRI images were acquired in a block design (repetition time [TR] = 1,000 ms, echo time [TE] = 37 ms, multi‐band acceleration factor 4, field of view 220 mm × 220 mm, 56 slices, acquisition voxel size 2.8 × 2.8 × 2.5 mm); 24 s of movement (green cues) alternated with 24 s of rest (red cues in same shapes but no movement) separated by periods of fixation (white cross for 8 s). Cue duration (2 s) and the inter‐trial interval (varied between 2.0 and 3.5 s) were the same as during practice in the laboratory. During all runs, a researcher observed hand movement and documented responses (right or left movement) to determine the accuracy of the movement direction. On both days, four functional MRI runs were completed, two in the Select condition and two in the Execute condition in alternating order; the condition completed in the first run (Select or Execute) was counterbalanced across participants. Each run included 2 movement blocks (24 s, 5 movement trials) for a total of 10 movement trials per run and lasted 2 min and 35 s.

On Day 1, structural brain images were also acquired and included T1 (TR = 2,250 ms, TE = 4.11 ms, 192 slices, 1 mm^3^ isotropic voxels) and T2 (T2 = 3,200 ms, TE = 567 ms, 176 slices, 1 mm^3^ isotropic voxels) structural scans for lesion identification and normalization of functional images. Diffusion‐weighted images were collected using an echo planar sequence (TR = 3,839 ms, TE = 71 ms, 68 slices, 1.8 mm^3^ isotropic voxels, 56 non‐collinear directions, *b* = 1,000 s/mm^2^) and used for determination of corticospinal tract (CST) integrity. Two runs of diffusion images were acquired with reverse encoding directions (anterior to posterior and posterior to anterior); seven b0 volumes were acquired in each run.

### 2.4 Data analysis

#### 2.4.1 Behavioural data

Data from the joystick during practice in the laboratory were used to determine movement direction (right/left), reaction time (RT), movement time, movement amplitude and peak velocity using a custom script in Matlab (Matworks, Inc., Natick, MA). Position data (*x*, *y*) were recorded throughout each trial (60 Hz) and used to derive movement velocity (Winter, 2005). Movement onset was defined as when velocity exceeded 5°/s for two consecutive samples or the change in velocity was very low (<1°/s) for two consecutive samples. Movement offset was defined as when velocity dropped below a minimum value (10°/s if peak velocity was <30; 25°/s if peak velocity was between 30 and 100; 40°/s if peak velocity was >100) after the time of peak velocity and either changed directions or the change in velocity was <5°/s. Movement planning performance was measured by movement direction accuracy (position at the time of peak velocity), RT (time between cue onset and movement onset; accurate trials only) and RT cost (Select RT–Execute RT). RT cost is a summary measure of the relative increase in planning time from the Execute to the Select condition that accounts for any effects of motor impairment on initiating a motor response and was a strong marker of Select performance in previous studies (Stewart et al., 2016; Stewart, Tran, & Cramer, 2014). Movement execution performance was measured by movement time (time between movement onset and movement offset), movement amplitude (total amplitude from movement onset to movement offset) and peak velocity. To determine the effect of practice on behavioural performance, a repeated measures analysis of variance was run on all movement variables with factors for condition (Execute, Select) and day. Movement direction accuracy was not normally distributed; therefore, movement direction accuracy was compared between Days 1 and 4 separately for each condition with a nonparametric Wilcoxon signed ranks test. Analysis of behavioral data was completed in SPSS 26 (IBM Corp., Armonk, NY) with a significance level of α < .05.

#### 2.4.2 Brain imaging data

All functional imaging data were analysed using SPM12 (Wellcome Trust Centre for Neuroimaging, London, UK). First, the origin of the structural T1 image was checked and repositioned to the anterior commissure as needed. Volumes from each functional MRI run were realigned and resliced to the first volume to account for motion artifact. Next, the realigned and resliced images were coregistered to the participant's structural T1‐weighted image. The participant's structural image was normalized to a T1 older brain template, with the stroke lesion masked out using the Clinical Toolbox (Rorden et al., 2012). The normalization parameters were then applied to all the realigned, resliced and normalized functional volumes for each run. The normalized images were resampled to 2 mm × 2 mm × 2 mm voxels and spatially smoothed with an isotropic Gaussian filter (full width at half maximum 8 mm); a temporal filter was then applied (1/128 Hz) to remove low frequency confounds. Data from each functional run were inspected for outliers due to excessive head motion (>2 mm translation or >0.2 radians rotation between each volume) or signal noise (*Z* > 5 from the mean image intensity) using the Artifact Detection Tool toolbox (http://www.nitrc.org/projects/artifact_detect); outliers were de‐weighted during statistical analysis (outliers averaged <1.3% of total volumes per day).

First‐level statistical analysis was performed separately for each participant using a general linear model (Friston, Holmes, Poline, et al., 1995; Friston, Holmes, Worsley, et al., 1995). For each run, movement and rest epochs were modeled separately against fixation for later contrast. Movement was contrasted with rest (move>rest) to determine the brain regions active during each condition (Execute, Select); both runs for each condition were weighted equally in all contrasts. The first derivative of head motion for all six directions was included as regressors of no interest to account for the effect of head motion in the data.

The contrast maps for each participant and each condition were moved to a second‐level random effects analysis with factors for condition (Execute, Select) and day (Day 1, Day 4); all data were flipped so that the left hemisphere was the lesioned side for group analyses. Both main effects (condition, day) and condition x day interactions were examined. Additionally, a *t* contrast between conditions (Select>Execute) was run for each day separately. Clusters were considered significant at *p* < .05 with a family‐wise error correction for multiple comparisons and a minimum size of 10 voxels.

Whole‐brain regression was completed to examine the relationship between Select behavioural performance and brain activation during Select. Analyses compared brain activation on Day 1 with RT cost on Day 1 to examine brain‐behaviour relationships at baseline and brain activation on Day 1 to RT cost on Day 4 to examine if activation patterns at baseline predicted behavioural performance at the end of practice. RT cost, the relative increase in RT for each individual for the Select condition relative to the Execute condition, was chosen as the behavioural measure for this analysis based on previous studies (Stewart et al., 2016; Stewart, Tran, & Cramer, 2014). Both positive relationships (higher brain activation relates to higher RT cost) and negative relationships (higher brain activation relates to lower RT cost) were examined. Age and movement time were included as regressors of no interest. For regression analyses, given the population and sample size, clusters were considered significant at a more liberal *p* < .001 uncorrected for multiple comparisons with a minimum size of 10 voxels.

An exploratory analysis of motor action selection task performance and PMd activation based on side of lesion was performed. We focused on PMd as this region has been shown to be a key node in the action selection network (Johansen‐Berg et al., 2002; O'Shea et al., 2007; Schluter et al., 1998). Percent signal change during Execute and Select was extracted from 4 mm radius regions‐of‐interest from ipsilesional PMd (MNI coordinates: −28‐12 68) and contralesional PMd (MNI coordinates: 32‐4 52) using MarsBaR (Brett et al., 2002); ipsilesional primary motor cortex (MNI coordinates: −34‐26 52) was also investigated. Behavioural performance on Days 1 and 4 (RT and RT cost) and brain activation from each region were compared between groups with a *t* test with the significance level set at α < .05.

Diffusion weighted images were analysed in FSL (FMRIB Center, Oxford, UK) using the FDT toolbox (Behrens et al., 2003) to quantify corticospinal tract integrity. Images were corrected for eddy currents and head motion followed by removal of the skull and dura (Smith, 2002). A voxelwise map of fractional anisotropy (FA) was then created. To determine corticospinal tract integrity, a region of interest mask was manually drawn on the three contiguous axial slices that showed the largest cross‐sectional area of the cerebral peduncle (Burke et al., 2014; Mark et al., 2008). This region was remote from the stroke lesion across participants and represents the integrity of the residual descending tract. Mean FA was extracted from each mask using a threshold of FA > 0.2, and an FA ratio (lesioned FA/nonlesioned FA) was calculated for each individual with a value of <1 indicating lower FA on the ipsilesional side relative to the contralesional side.

## 3 RESULTS

### 3.1 Participants

**FIGURE 2:**
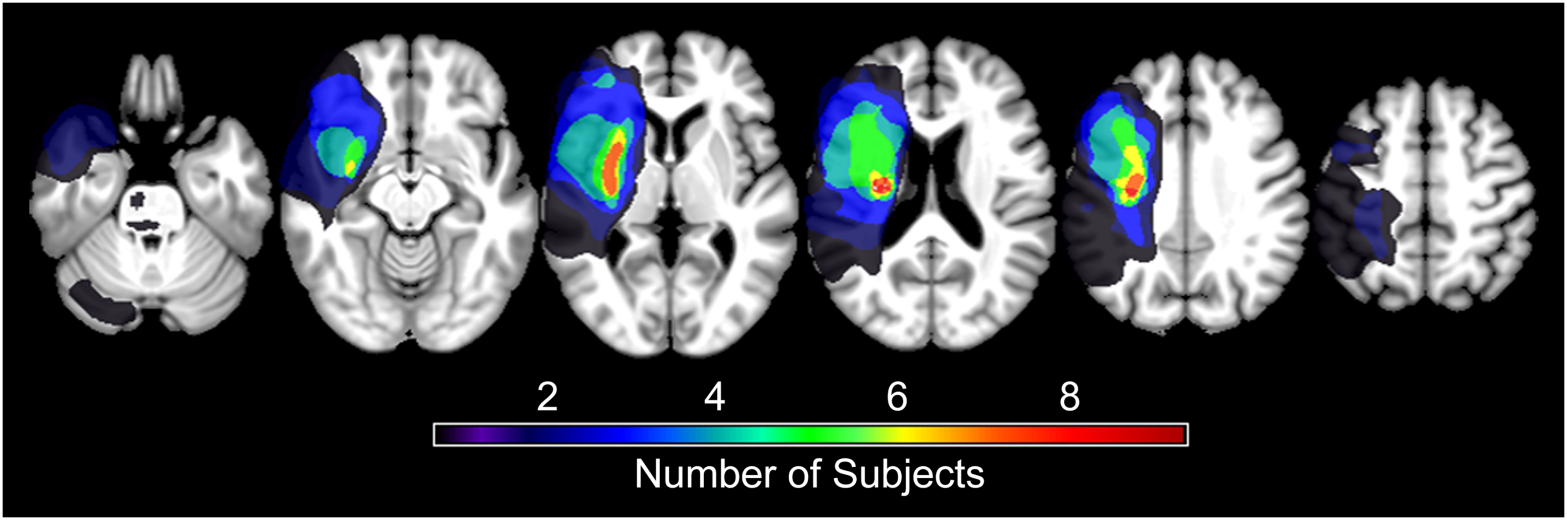
Summary mask of stroke lesions. All lesions were flipped to the left side. Colour represents number of participants with a lesion in that voxel. Note that the cerebellar lesion was from a previous stroke in a single participant.

Overall, individuals presented with mild to moderate motor impairment (mean UE FM motor score of 43.3, range 18–61), reported continued difficulty in using the weaker hand to complete functional activities (mean Stroke Impact Scale Hand Domain score of 44.4, range 5–100) and did not have apraxia (Test for Upper Limb Apraxia scores all >194) (Table 1). Participants had a mix of subcortical and cortical lesions (Figure 2) that were distributed between the right and left sides (Table 1). One participant had a previous stroke in the right cerebellum (Subject ID 7); however, this lesion did not significantly impact movement in the tested hand (left). As expected, CST FA was lower in the lesioned hemisphere (mean = 0.594, range 0.428–0.700) compared to the nonlesioned hemisphere (mean = 0.661, range 0.547–0.739) leading to a mean FA ratio of 0.90 (range 0.76–1.1).

**TABLE 1:** Participant demographics

Subject ID	Sex	Age	Months post‐stroke	Lesion side	Lesion volume (cc)	CST FA ratio	TULIA (max 260)	UE FM (max 66)	BBT (more/less)	SIS hand domain (max 100)
1	M	54	64	L	187.4	1.0	229	54	37/48	50
2	M	67	158	L	113.2	0.96	238	51	50/51	60
3	F	63	49	L	3.1	0.93	240	47	22/34	45
4	M	65	113	L	67.7	0.88	221	47	33/49	35
5	M	59	25	R	1.7	0.84	235	20	3/45	5
6	M	61	41	L	0.2	0.84	224	26	13/42	15
7	M	40	53	R	23.0	0.92	236	61	48/51	95
8	M	56	13	R	15.0	0.76	237	24	8/53	50
9	M	47	11	R	3.3	0.88	237	21	4/68	5
10	M	61	12	L	0.2	1.1	232	52	44/50	100
11	M	57	6	L	1.6	0.89	234	36	28/46	30
12	F	53	13	R	30.0	0.94	217	43	23/31	15
13	F	60	7	R	3.7	0.99	231	59	44/48	75
14	F	35	41	L	0.8	0.88	240	59	16/51	45
15	F	64	12	R	16.3	0.84	231	54	36/44	70
16	M	56	22	L	1.0	0.78	236	18	2/45	15
Mean	11 M/5F	56.1	40.0	9 L/7R	29.3	0.90	232.4	43.3	25.7/47.3	44.4

Abbreviations: CST FA Ratio, corticospinal tract fractional anisotropy ratio (ipsilesional FA/contralesional FA); TULIA, test for upper limb apraxia; UE FM, upper extremity Fugl–Meyer motor score; BBT, box and blocks test (more impaired arm/less impaired arm); SIS, stroke impact scale; M, male; F, female; L, left; R, right.

### 3.2 Motor action selection task performance

**FIGURE 3:**
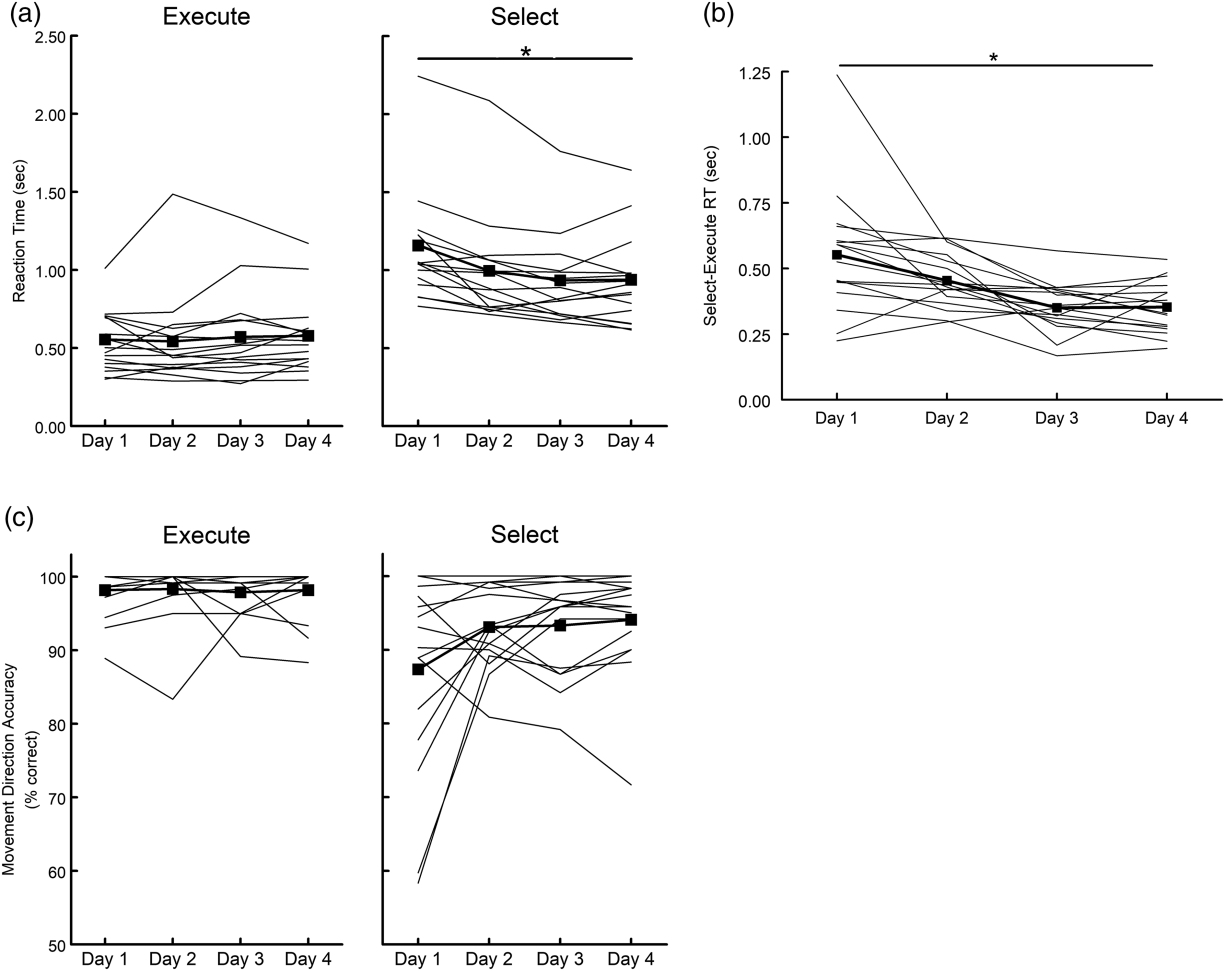
Motor planning performance on the motor action selection task for both conditions across days for reaction time (a), reaction time cost (b, Select RT‐Execute RT) and movement direction accuracy (c). Each thin line represents an individual participant; squares represent the group average. *p < .02

On Day 1, Execute performance was highly accurate (98.2 ± 3.3%), and mean reaction time averaged 0.542 ± 0.207 s (Figure 3). As expected, movement direction accuracy was lower (87.3 ± 13.6%; *p* = .002) and RT was longer (1.11 ± 0.362 s; *p* < .001) for the Select condition compared to the Execute condition. However, over days of practice, performance in the Select condition improved for both movement direction accuracy (*p* = .06) and RT (*p* = .02); Execute performance did not change with practice (*p* > .397). RT cost also decreased with practice from 0.552 ± 0.238 s on Day 1 to 0.353 ± 0.099 s on Day 4 (*p* = .002). One participant had a relatively high RT cost on Day 1 (greater than 2.5 standard deviations higher than the group mean; Figure 3b). Analyses of RT cost and Select RT were repeated with this participant removed; both variables still showed a significant decrease over days (RT cost: *p* = .004; Select RT: *p* = .02). Measures of movement execution (movement time, movement amplitude and peak velocity) did not differ between conditions (*p* > .095) and did not change with practice (*p* > .616; Figure S1). Age, months post‐stroke, level of motor impairment (UE FM), movement amplitude and CST FA ratio did not correlate with RT cost on Day 1 or 4 (*r* < .343, *p* > .194). Additionally, RT cost on Day 1 (baseline action selection performance) did not significantly predict RT cost on Day 4 (*r* = .368, *p* = .160). CST FA ratio did correlate with movement time during Select on Day 4 (*r* = −.639, *p* = .008) but not on Day 1 (*r* = −.345, *p* = .190), while UE FM did not correlate with movement time on either day (*r* < .291, *p* > .275).

During functional MRI, movement direction accuracy was quantified by visualization of movement. For the Execute condition, movement direction accuracy was high on Day 1 (98.1 ± 7.5) and Day 4 (98.4 ± 4.0). For the Select condition, movement direction accuracy during scanning increased slightly over days (*p* = .048) from 90.3 ± 15.1 on Day 1 to 94.7 ± 10.6 on Day 4.

### 3.3 Effect of practice on brain activation during action selection

**FIGURE 4:**
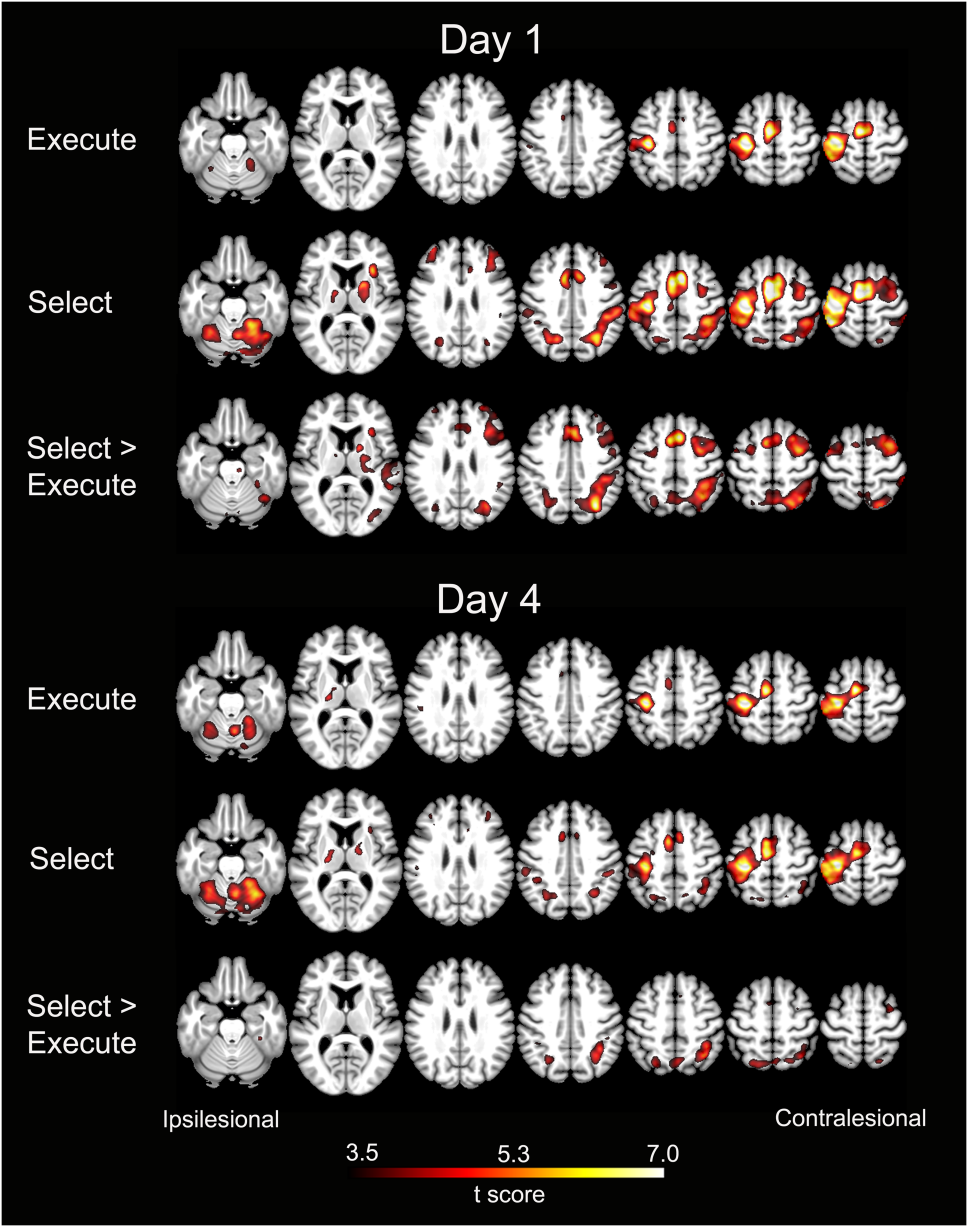
Group activation maps. Execute and Select activation shown compared to rest shown for Days 1 and 4. Select>Execute represents the *t* contrast between conditions separately for each day.

Brain activation during both the Execute and Select conditions across days is shown in Figure 4. On Day 1, activation during the Execute condition was in the expected motor network including ipsilesional primary motor cortex, sensory cortex, PMd, supplemental motor area (SMA) and contralesional cerebellum (Figure 4; Table S1). Activation during the Select condition included a network of brain regions in both hemispheres; relative to the Execute condition (Select>Execute), activation during the Select condition was higher in several regions in the action selection network including PMd, SMA, anterior cingulate cortex (ACC) and parietal cortex (Table 2). Overall, activation increases to meet the demands of the Select condition were greater in the contralesional hemisphere.

**TABLE 2:** Location of significant clusters between conditions

			MNI coordinates		
Cluster volume	Brain region	Peak T	*x*	*y*	*z*
Select>Execute Day 1					
458	CL precuneus	7.02	30	−68	38
	CL superior parietal lobule	5.97	34	−58	50
	CL inferior parietal lobule	5.53	34	−44	46
327	CL supplementary motor area	6.92	4	16	48
	IL supplementary motor area	6.40	−8	10	50
	CL anterior cingulate cortex	6.34	6	20	40
64	CL insula	6.11	30	24	2
267	CL dorsal premotor cortex	5.92	32	−2	52
	CL superior frontal gyrus	5.27	24	14	54
70	CL superior parietal lobule	5.87	24	−66	54
15	CL superior temporal gyrus	5.52	60	−44	18
25	CL superior temporal gyrus	5.29	54	−30	18
11	CL inferior parietal lobule	5.23	42	−36	36
Select>Execute Day 4					
76	CL inferior parietal lobule	5.43	36	−56	48
Day x condition interaction		Peak F			
10	IL anterior cingulate cortex	30.09	−2	28	36

*Note*: All clusters were significant at *p* < .05 with familywise error correction. For larger clusters, the locations of several local maxima within the clusters are listed.

Abbreviations: cluster volume, number of 8 mm^3^ voxels in cluster; peak T, peak *t* value within the cluster; IL, ipsilesional; CL, contralesional.

On Day 4, after 4 days of behavioural practice, a similar network was activated during the Select condition (Figure 4; Table S2); relative to Execute (Select>Execute comparison), however, the only brain region with significantly increased activation during Select was contralesional parietal cortex (Table 2). A significant day x condition interaction was found for a region in the ACC (Table 2). On Day 1, activation in this brain region increased from Execute to Select, but, on Day 4, activation decreased slightly in Select compared to Execute (Figure S2). No additional clusters were found for the day x condition interaction or the main effect of day at a corrected *p* < .05.

### 3.4 Neural correlates of action selection performance

**FIGURE 5:**
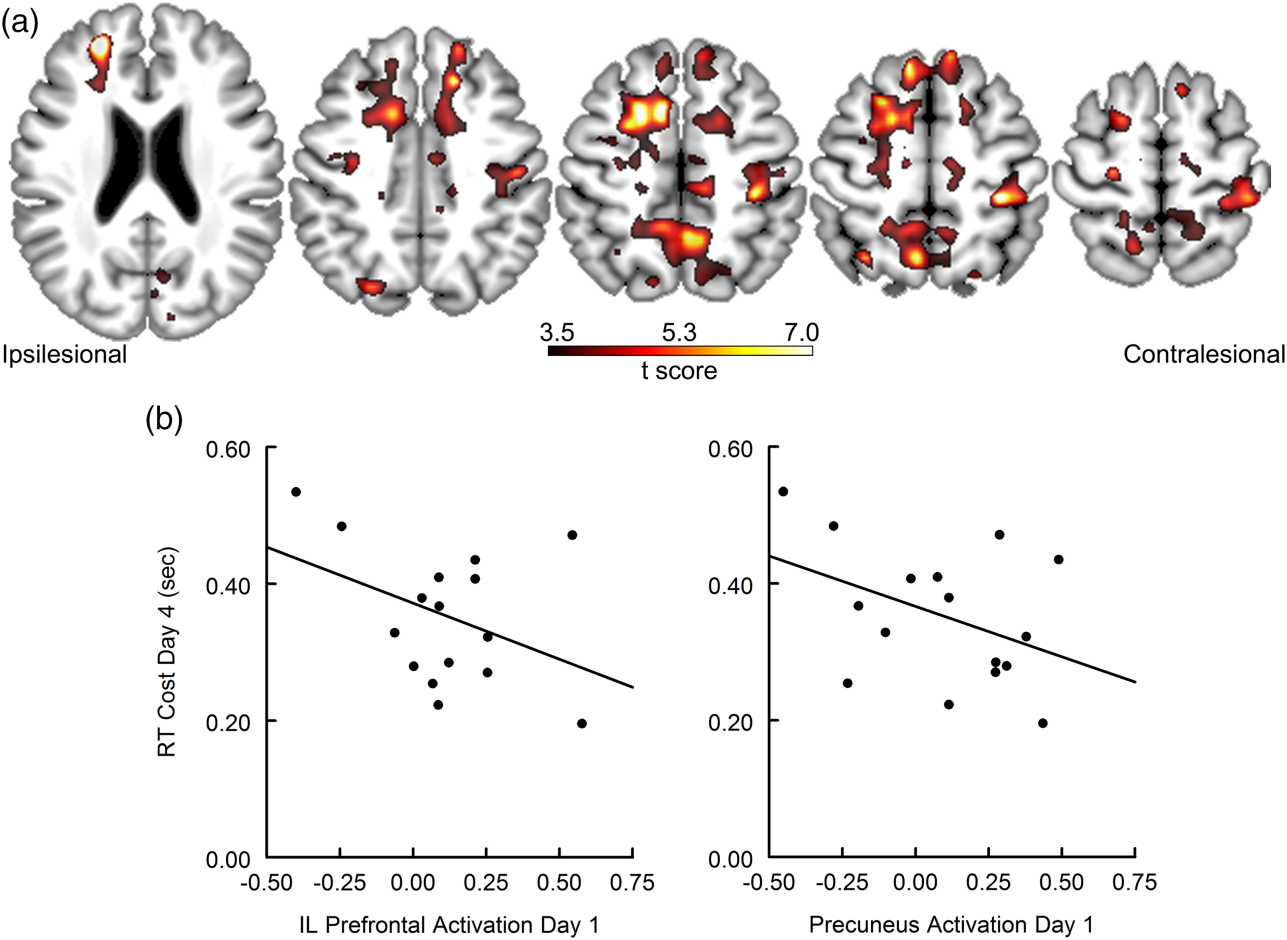
Results of whole‐brain regression between brain activation during Select on Day 1 and RT cost on Day 4 (a) with age and MT included as regressors of no interest (negative correlation at *p* < .001 uncorrected for multiple comparisons). Percent signal change was extracted from two significant clusters to visualize the relationship between brain activation on day 1 and RT cost on day 4 (b): Ipsilesional (IL) prefrontal cortices (dorsolateral prefrontal cortex, supplementary motor area and anterior cingulate cortex) and bilateral precuneus

Whole‐brain regression analyses were run to determine if Select performance related to brain activation during Select. At baseline, there was no significant relationship between brain activation during Select on Day 1 and RT cost on Day 1. However, activation during Select on Day 1 predicted RT cost on Day 4 (Figure 5; Table 3). Higher activation in several brain regions in both hemispheres on Day 1 predicted lower RT cost (better performance) on Day 4 including several regions of the ipsilesional and contralesional action selection network (SMA, ACC and precuneus). Additionally, individuals that had greater engagement of bilateral dorsolateral prefrontal cortices and contralesional sensory cortex on Day 1 showed better behavioural performance on Day 4. A follow‐up analysis examined whether higher brain activation during Execute on Day 1 also predicted RT cost on Day 4; a single cluster was found in the contralesional sensory cortex (MNI coordinates: 42‐30 56; 277 voxels; peak *T* = 5.24). There was no significant positive relationship between baseline brain activation and RT cost on Day 4 (i.e., higher activation predicted higher RT cost).

**TABLE 3:** Location of significant clusters for regression analysis

			MNI coordinates		
Cluster volume	Brain region	Peak T	*x*	*y*	*z*
Select activation on day 1 with RT cost on day 4 (−)					
1,401	IL dorsolateral prefrontal cortex	16.67	−24	44	22
	IL supplementary motor area	8.60	−10	10	46
	IL anterior cingulate cortex	5.83	−4	26	34
	CL anterior cingulate cortex	5.35	4	14	28
	IL superior frontal gyrus	5.20	−24	10	54
468	CL sensory cortex	8.86	34	−30	50
608	CL anterior cingulate	7.06	16	26	40
	CL dorsolateral prefrontal cortex	5.85	20	44	34
541	IL precuneus	6.90	−6	60	50
	CL precuneus	6.76	4	−50	46

*Note*: All clusters were significant at *p* < .001 uncorrected for multiple comparisons. For larger clusters, the locations of several local maxima within the clusters are listed.

Abbreviations: cluster volume, number of 8 mm^3^ voxels in cluster; peak T, peak *t* value within the cluster; IL, ipsilesional; CL, contralesional. RT cost, reaction time cost; (−), negative relationship.

### 3.5 Side of lesion and action selection performance

**FIGURE 6:**
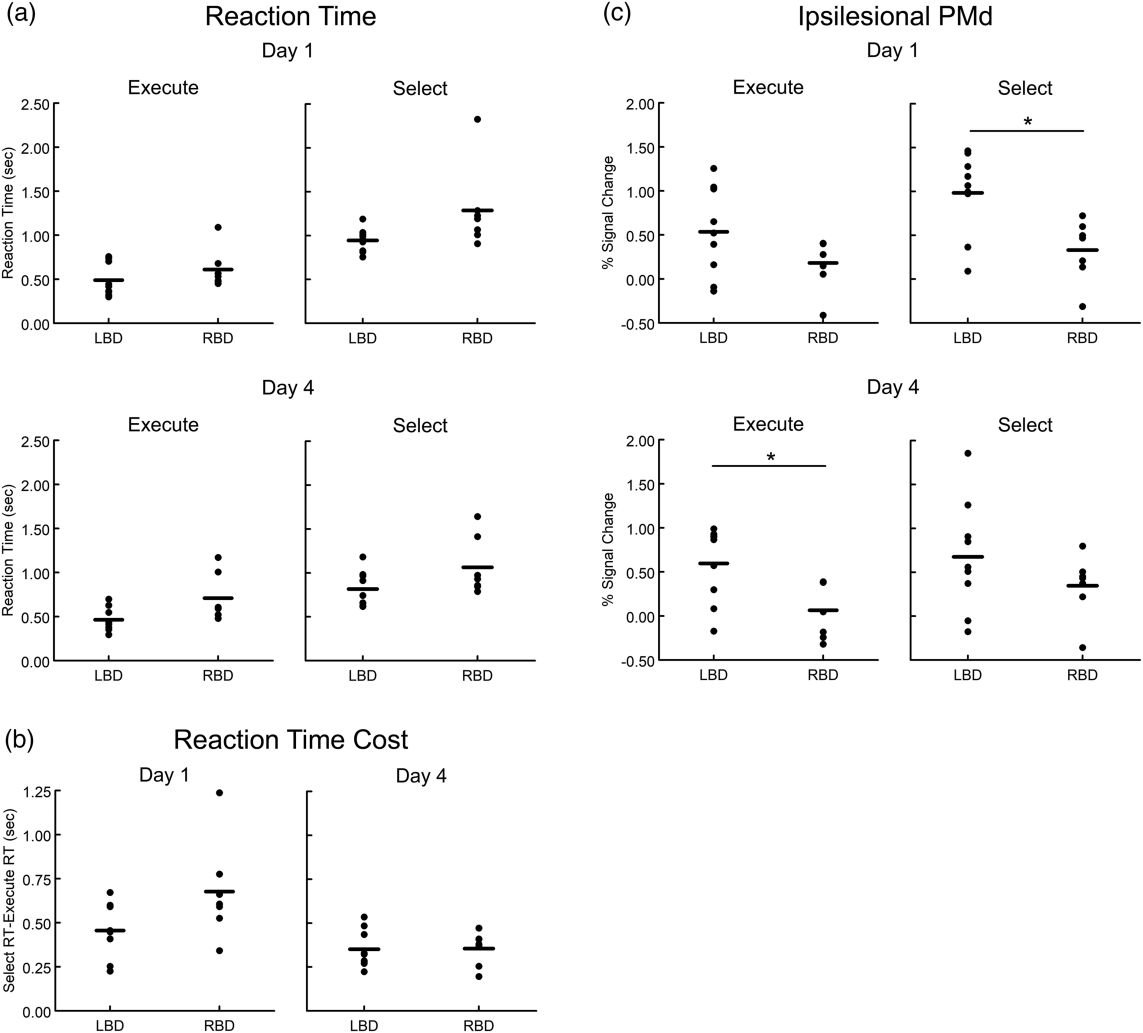
Comparisons based on side of lesion on Days 1 and 4 for reaction time (a) and reaction time cost (b, Select RT‐Execute RT) during behavioural practice and percent signal change in ipsilesional dorsal premotor cortex (c) during functional MRI. Each circle represents an individual participant, center line = mean. LBD, left brain damage; RBD, right brain damage; IL PMd, ipsilesional dorsal premotor cortex. *p < .02

Individuals were divided into subgroups based on side of lesion: left brain damage (LBD; *N* = 9) and right brain damage (RBD; *N* = 7) (see Figure S3 for lesion distribution by subgroup). Overall, on Days 1 and 4 of behavioural practice, individuals with RBD who performed the action selection task with the nondominant, left hand had longer reaction times than individuals with LBD who performed the task with the dominant, right hand (Figure 6); these differences approached statistical significance for Select RT on Day 1 (*p* = .057) and Execute RT on Day 4 (*p* = .056). RT cost was also higher on Day 1 in the RBD subgroup compared to the LBD subgroup, but this difference did not reach statistical significance (*p* = .062); on Day 4, RT cost was similar between the two subgroups (*p* = .946). This higher RT in the RBD subgroup corresponded to lower ipsilesional PMd activation relative to the LBD subgroup (Figure 6c) with statistically significant differences during Select on Day 1 (*p* = .008) and Execute on Day 4 (*p* = .016). There were no significant differences in contralesional PMd or ipsilesional primary motor cortex activation based on side of lesion (*p* > .158 for all comparisons). RBD and LBD subgroups did not significantly differ on level of motor impairment (UE FM motor score: LBD = 45.6, RBD = 40.3; *p* = .525), CST FA ratio (LBD = 0.92, RBD = 0.88; *p* = .437) or Test for Upper Limb Apraxia score (LBD = 232.7, RBD = 232.0; *p* = .852).

## 4 DISCUSSION

This study examined the behavioural and neural correlates of behavioural practice targeted at the action selection network after stroke. At baseline (Day 1), compared to Execute, the Select condition led to a longer reaction time and a corresponding increase in brain activation, especially in the contralesional hemisphere. After 4 days of practice, Select performance improved (faster reaction time) while brain activation to complete the Select condition was reduced, including in the contralesional hemisphere. Additionally, brain activation at baseline predicted behavioral performance at the end of the practice. Individuals who had higher levels of activation in ipsileisonal and contralesional regions of the action selection network, prefrontal cortex and contralesional sensory cortex at baseline had better behavioral performance (lower RT cost) at the end of practice. Overall, the results of the current study suggest that behavioural practice can improve action selection performance with a corresponding reduction in brain activation to successfully complete this motor preparation process. Systematic changes in task practice conditions with predictable behavioural and neural effects may be useful in designing novel rehabilitation interventions after stroke (Stewart et al., 2020).

The added action selection demands in the Select condition on Day 1 corresponded to an increase in brain activation primarily in the contralesional hemisphere that was ipsilateral to the moving hand similar to a previous study in stroke (Stewart et al., 2016). Brain regions engaged during Select were consistent with areas reported to be part of the action selection network in nondisabled individuals including PMd, SMA, ACC and inferior parietal lobule (Grafton et al., 1998; Grol et al., 2006; O'Shea et al., 2007; Toni et al., 2002). While activation in these regions was present in both hemispheres in the current study, the relative increase in activation for Select compared to Execute was greater in the contralesional hemisphere. Engagement of the contralesional hemisphere to meet an increase in task demands completed with the more impaired hand has been reported previously in individuals post‐stroke for tasks with increased cognitive demands (Dennis et al., 2011) and increased coordination demands (Schaechter & Perdue, 2008). In the current study, we found a similar contralesional response to an increase in action selection demands primarily in brain regions that are part of the action selection network.

After 4 days of practice, individuals were able to complete action selection with a shorter RT that corresponded to less brain activation throughout the action selection network relative to execution alone; only the contralesional parietal cortex showed an increase in activation in Select compared to Execute on Day 4. This continued activation in parietal cortex to complete action selection may be related to the distinct role the parietal cortex plays in the selection process (stimulus–response mapping) compared to other brain regions (e.g., premotor cortex and movement planning) (Cavina‐Pratesi et al., 2006). Practice‐related changes in brain activation in young, nondisabled individuals have not been reported as behavioural performance for action selection in that population has been shown to be stable over days (O'Shea et al., 2007). In individuals post‐stroke, as behavioural performance improved with practice (reduced planning time), the modulation of brain activation from simple movement (Execute) to action selection (Select) was reduced. Overall, the reduction in modulation of brain activation for Select was likely related to the reduction in response duration (i.e., shorter reaction times) (Grinband et al., 2011) and was present throughout the action selection network, especially in the contralesional hemisphere. This finding suggests that the contralesional activation seen in response to an increase in task demands after stroke (Dennis et al., 2011; Schaechter & Perdue, 2008; Stewart et al., 2016) may be modifiable with practice. Practice conditions that include more complex aspects of skilled movement or engage a specific movement related network, such as action selection, may provide a challenging practice environment that benefits outcomes.

PMd is a key node in the action selection network (Grafton et al., 1998; Grol et al., 2006; O'Shea et al., 2007; Toni et al., 2002) and was engaged during Select at baseline in the current study. PMd is active during a variety of movement preparation processes, including action selection, however, this region also supports movement execution through contributions to descending motor tracts and connections with primary motor cortex (Archer et al., 2018; Bestmann et al., 2008; Dum & Strick, 2002; Picard & Strick, 2001). After stroke, compensatory activation in PMd during movement has often been reported (Bestmann et al., 2010; Fridman et al., 2004; Johansen‐Berg et al., 2002; Ward et al., 2006), however, it is not clear if this activation is supporting movement planning or movement execution. PMd may serve as a region of interaction between the motor and cognitive systems (Abe & Hanakawa, 2009; Hanakawa, 2011). The ability to flexibly allocate PMd resources to either motor planning or movement execution may have an impact on the performance of complex, functional tasks (Maes et al., 2020). At the end of practice, individuals in the current study did not show a significant increase in PMd activation to complete action selection which may allow this neural resource to be used for other aspects of skilled movement control.

Brain activation at baseline did not correlate with behavioural performance at baseline but did predict RT cost at the end of practice. A previous cross‐sectional study found a positive correlation between ipsilesional PMd and prefrontal activation and RT cost (Stewart et al., 2016). However, that analysis included both individual's post‐stroke and age‐matched controls and did have the ability to predict performance after a period of practice. In the current study, higher brain activation during action selection on Day 1 in several regions of the ipsilesional and contralesional action selection network (SMA, ACC, superior frontal gyrus and precuneus) as well as bilateral dorsolateral prefrontal cortex and contralesional sensory cortex predicted better behavioral performance on Day 4 (lower reaction time cost). Baseline behaviour performance and clinical measures of the motor system were not significant predictors. Overall, this finding suggests that individuals who had greater engagement of the action selection network and cognitive brain regions early in practice, despite their initial behavioral performance level, had better action selection performance at the end of practice. Baseline measures of brain function have been shown to predict response to a period of motor practice after stroke (Zhou et al., 2018). In the current study, we show a similar, network specific relationship for practice‐related changes on a motor‐cognitive task that has action selection demands.

A previous study on the neural correlates of action selection with the more impaired hand after stroke only included right‐hand dominant individuals with left‐sided brain damage (Stewart et al., 2016) due to the hypothesized specialized role the left hemisphere is thought to play in action selection (Cavina‐Pratesi et al., 2006; Rushworth et al., 1998; Schluter et al., 1998). However, both the left and right PMd can support action selection (O'Shea et al., 2007), and stroke lesions occur in both the right and left hemispheres with residual motor deficits presenting in the left arm as well as the right arm. Therefore, understanding the effect of practice on the behavioural and neural correlates of action selection after left‐ and right‐sided brain damage is needed to translate results to the broader stroke population. While the current study was underpowered to perform a whole brain analysis to allow full assessment of differences based on side of brain damage, the results of the exploratory analysis suggest that the behavioral and neural correlates of action selection may differ in individuals with right‐brain damage and need further investigation in a larger study population.

Performance differences between Execute and Select and changes with practice were found in measures of movement planning (RT cost and Select RT) but not measures of movement execution (movement time, movement amplitude and peak velocity). This finding is consistent with the structure of the experimental task as movement planning demands differed between conditions but movement execution demands did not. This finding may also explain why level of motor impairment or CST FA ratio did not correlate with the primary measure of behavioral performance (RT cost) as these measures may be more closely aligned with measures of movement execution after stroke. Additionally, the task used in the current study did not include spatial or temporal demands for movement execution beyond a general movement to the right or left. A similar task with greater demands for movement execution (e.g., the accuracy demands of capturing a target) may impact planning performance (Orban de Xivry et al., 2017) or have different effects based level of motor impairment or degree of CST integrity. Future studies could build on the findings of the current study by investigating the effect of practice on tasks with varied demands after stroke.

Movement performance data from the joystick during functional MRI were not consistently available due to difficulty with positioning (*N* = 3) and technical issues with data collection (*N* = 4) in several participants. While movement direction accuracy data were available for all participants, reaction time and movement time collected during scanning would have been optimal. However, a previous study in individuals post‐stroke using the same paradigm found that the primary behavioral measure, RT cost, was similar between behavioural performance just prior to scanning and behavioural performance during scanning (Stewart et al., 2016). Previous studies have shown a relationship between movement amplitude and brain activation (Fabbri et al., 2012; Waldvogel et al., 1999). Although movement amplitude and other measures of movement execution that are often related to movement amplitude (movement time and peak velocity) did not significantly differ between conditions or change over days, movement amplitude was not controlled in the experimental task. Post hoc analyses on behavioral data collected in the laboratory on Days 1 and 4 found that movement amplitude during Execute correlated with movement amplitude during Select (*r* > .677, *p* < .004), suggesting that individual participants had similar movement amplitudes in the two conditions. However, we can not fully rule out the impact of movement amplitude on brain activation between conditions. The block design used in the current study limited the ability to consider response accuracy and individual reaction times in the analysis of brain data. While mean direction accuracy was relatively high during Select during scanning (>90% both days), response accuracy was lower than during Execute. The inclusion of both accurate and inaccurate trials may have impacted the activation patterns seen over days and conditions. Additionally, the modelling of individual reaction times was not possible which can impact the brain activation patterns seen (Grinband et al., 2008). This study included individuals with mild to moderate motor impairment in the chronic phase of stroke recovery with relatively high CST FA ratios. The findings of this study may not apply to individuals who present more severe motor impairment, are in the acute or subacute phase of recovery or have a lower relative level of CST integrity. Additionally, our sample size did not provide sufficient power to examine whether changes in behavioural performance or brain activation with practice differed based on level of motor impairment (e.g., moderate versus mild). It is possible that activation in the contralesional hemisphere was related to mirror movements of the less impaired hand (Soteropoulos et al., 2011). We did not fully assess for mirror movements during scanning; however, activation ipsilateral to the moving hand has been shown in individuals without stroke in challenging conditions where the effect of mirror movements have been controlled (Barany et al., 2020; Buetefisch et al., 2014). Individuals in this study did not present with significant apraxia, and medications were not monitored. Action selection performance and the behavioural and neural response to a period of practice may be different in individuals with apraxia or who are taking medications that may impact motor learning.

In conclusion, individuals with mild to moderate motor impairment post‐stoke improved performance on an action selection task after 4 days of practice. Action selection performance improved (faster reaction time) while brain activation to complete the action selection was reduced, especially in the contralesional hemisphere. Individuals who had higher levels of activation in ipsileisonal and contralesional regions of the action selection network, prefrontal cortex and contralesional sensory cortex at baseline had better behavioural performance at the end of practice. The results of the current study suggest that behavioural practice can improve action selection performance and reduce the brain activation required to successfully complete this motor preparation process. Systematic changes in behavioral practice, such as adding action selection demands, may allow the targeting of specific components of the motor network during rehabilitation after stroke.

## CONFLICT OF INTEREST

The authors declare no financial or other conflict of interest.

## AUTHOR CONTRIBUTIONS

Jill Campbell Stewart contributed to the conceptualization, formal analysis, funding acquisition, methodology, investigation, visualization and writing‐original draft. Jessica F. Baird contributed to the formal analysis, methodology, investigation and writing‐review and editing. Allison F. Lewis contributed to the formal analysis, methodology, investigation and writing‐review and editing. Stacy L. Fritz contributed to the conceptualization, funding acquisition and writing‐review and editing. Julius Fridriksson contributed to the conceptualization, funding acquisition and writing‐review and editing.

### PEER REVIEW

The peer review history for this article is available at https://publons.com/publon/10.1111/ejn.15754.

## Supporting information


**Figure S1.** Movement execution performance for the motor action selection task for both condition across days for movement time (A), movement amplitude (B), and peak velocity (C). Variables did not differ between conditions or change over days. Each thin line represents an individual participant; squares represent the group average.
**Figure S2.** Significant cluster in ipsilesional anterior cingulate cortex (MNI coordinates: −2 28 36) that showed a significant day x condition interaction (*p* < 0.05 with FWE correction for multiple comparisons). Percent signal change was extracted from the cluster; activation increased from Execute to Select on Day 1 but decreased from Execute to Select on Day 2.
**Figure S3.** Summary mask of stroke lesions by side of brain damage (LBD = Left Brain Damage; RBD = Right Brain Damage). Color represents number of participants with a lesion in that voxel. Note that the cerebellar lesion in the RBD group was from a previous stroke in a single participant.
**Table S1.** Location of significant clusters on Day 1
**Table S2.** Location of significant clusters on Day 4

## Data Availability

The data that support the findings of this study are available from the corresponding author upon reasonable request.
