# Supplementary material for: Effect of behavioural practice targeted at the motor action selection network after stroke
Source: Eur J Neurosci. 2022 Jul 17;56(4):4469–85. doi: 10.1111/ejn.15754 (PMC9380182; doi:10.1111/ejn.15754)
Supplement: Supplementary file 1 — Figure S1. Movement execution performance for the motor action selection task for both condition across days for movement time (A), movement amplitude (B), and peak velocity (C). Variables did not differ between conditions or change over days. Each thin line represents an individual participant; squares represent the group average. Figure S2. Significant cluster in ipsilesional anterior cingulate cortex (MNI coordinates: −2 28 36) that showed a significant day x condition interaction (p < 0.05 with FWE correction for multiple comparisons). Percent signal change was extracted from the cluster; activation increased from Execute to Select on Day 1 but decreased from Execute to Select on Day 2. Figure S3. Summary mask of stroke lesions by side of brain damage (LBD = Left Brain Damage; RBD = Right Brain Damage). Color represents number of participants with a lesion in that voxel. Note that the cerebellar lesion in the RBD group was from a previous stroke in a single participant. Table S1. Location of significant clusters on Day 1 Table S2. Location of significant clusters on Day 4 [file EJN-56-4469-s001.pdf]

Supplementary Data

Effect of behavioral practice targeted at the motor action selection network after stroke

Jill Campbell Stewart[1], Jessica F. Baird[1], Allison F. Lewis[1],
Stacy L. Fritz[1], Julius Fridriksson[2]

[1]Department of Exercise Science, University of South Carolina
[2]Department of Communication Sciences & Disorders, University of South Carolina

Corresponding Author: Jill Campbell Stewart, PT, PhD
University of South Carolina
921 Assembly Street, Room 301E
Columbia, SC 29208
PHONE: (803) 777-6583
FAX: (803) 777-0558
EMAIL: jcstewar@mailbox.sc.edu

Supplemental Figure 1.

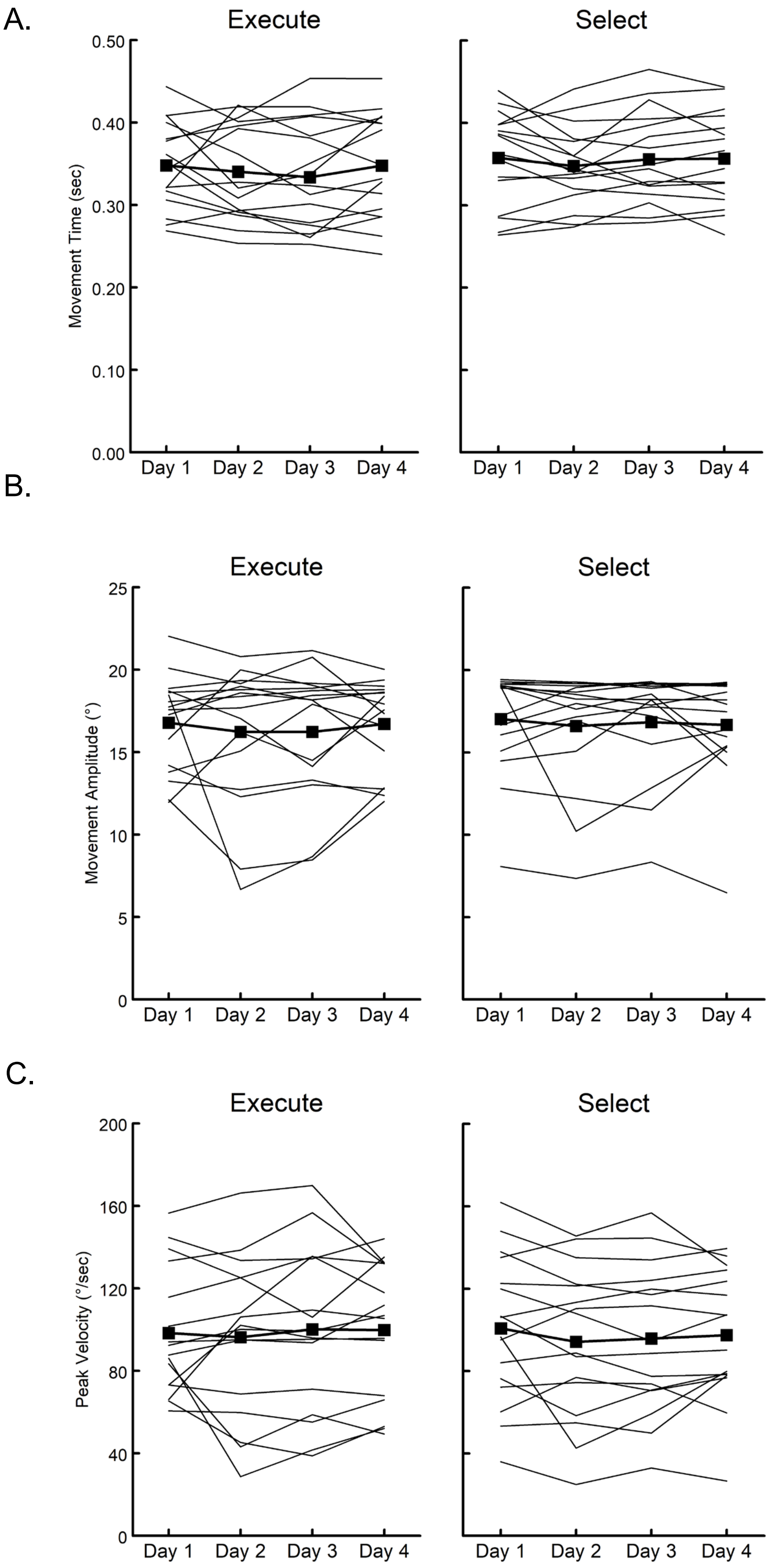

Supplemental Figure 1. Movement execution performance for the motor action selection task for both condition across days for movement time (A), movement amplitude (B), and peak velocity (C). Variables did not differ between conditions or change over days. Each thin line represents an individual participant; squares represent the group average.

Supplemental Figure 2.

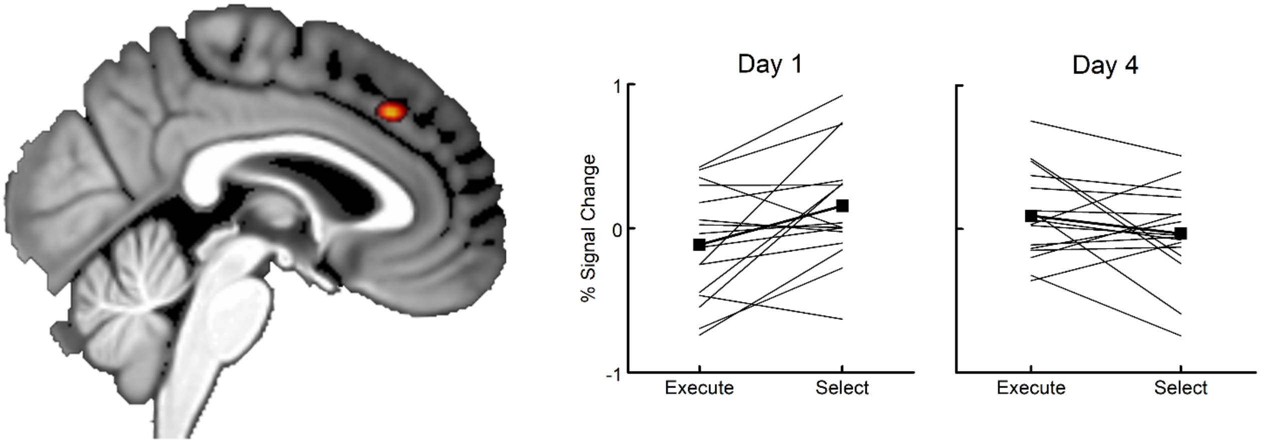

Supplemental Figure 2. Significant cluster in ipsilesional anterior cingulate cortex (MNI coordinates: -2 28 36) that showed a significant day x condition interaction (p<0.05 with FWE correction for multiple comparisons). Percent signal change was extracted from the cluster; activation increased from Execute to Select on Day 1 but decreased from Execute to Select on Day 2.

Supplemental Figure 3.

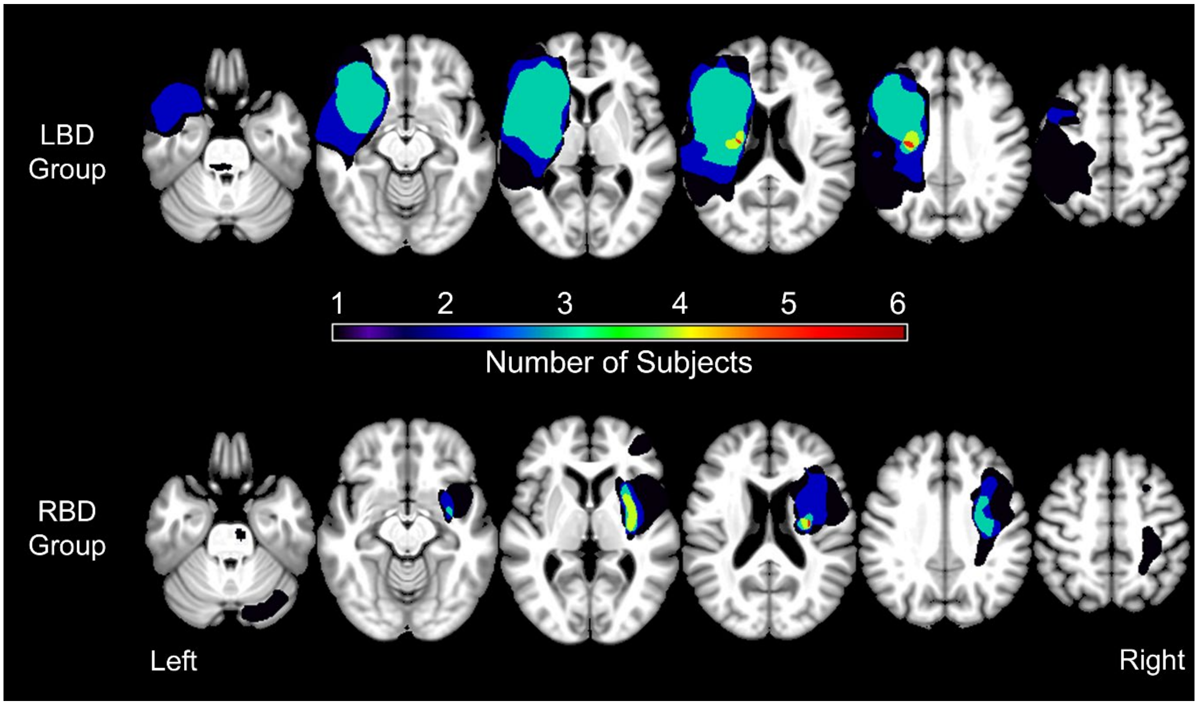

Supplemental Figure 3. Summary mask of stroke lesions by side of brain damage (LBD=Left Brain Damage; RBD=Right Brain Damage). Color represents number of participants with a lesion in that voxel. Note that the cerebellar lesion in the RBD group was from a previous stroke in a single participant.

Supplemental Table 1. Location of significant clusters on Day 1

Condition	Cluster Volume	Brain region	Peak T	MNI coordinates x	y	z
Execute	1281	IL primary motor cortex	8.01	-34	-26	52
		IL dorsal premotor cortex	7.70	-28	-18	72
		IL sensory cortex	7.62	-42	-38	52
	325	IL supplementary motor area	7.81	-6	-10	56
	16	CL cerebellum	5.67	22	-48	-50
	17	CL cerebellum	5.40	10	-54	-14
Select	3713	IL supplementary motor area	8.67	-8	-8	56
		IL primary motor cortex	8.31	-34	-26	52
		IL sensory cortex	8.23	-42	-38	62
		IL dorsal premotor cortex	8.21	-28	-12	72
		IL supplementary motor area	8.01	-6	2	54
		Cl supplementary motor area	7.79	6	10	48
		IL anterior cingulate cortex	6.15	-8	6	42
		CL dorsal premotor cortex	5.88	22	-2	72
	779	CL cerebellum	6.26	10	-56	-14
	82	CL insula	6.60	32	18	12
	141	CL visual cortex	6.33	30	-62	36
	109	CL cerebellum	6.07	22	-48	50
	286	CL inferior parietal lobule	5.91	38	-42	50
	34	CL putamen	5.54	18	4	8
	15	CL inferior frontal gyrus	5.42	34	30	24
	13	IL precuneus	5.38	-18	-62	38
	21	IL inferior parietal lobule	5.31	-28	-62	40
	20	IL cerebellum	5.30	-28	-54	-22
	15	CL inferior parietal lobule	5.28	34	-56	48
	12	CL precuneus	5.27	18	-62	52
	11	IL cerebellum	5.25	-32	-44	-44
	16	CL dorsal premotor cortex	5.23	32	-4	52

All clusters were significant at $p<0.05$ with familywise error correction. Cluster volume=number of 8 mm^3 voxels in cluster; Peak T=peak t value within the cluster; IL=ipsilesional; CL=contralesional. For larger clusters, the locations of several local maxima within the clusters are listed.

Supplemental Table 2. Location of significant clusters on Day 4

Condition	Cluster Volume	Brain region	Peak T	MNI coordinates x	y	z
Execute	833	IL primary motor cortex	8.18	-30	-26	54
		IL sensory cortex	6.45	-44	-36	62
		IL dorsal premotor cortex	5.36	-28	-18	72
	170	IL supplementary motor area	7.67	-8	-10	56
	184	CL cerebellum	6.03	12	-48	-14
	15	CL cerebellum	5.22	20	-54	-48
Select	1271	IL primary motor cortex	7.89	-30	-26	54
		IL dorsal premotor cortex	7.35	-34	-20	68
		IL sensory cortex	6.59	-40	-38	54
	316	IL supplementary motor area	7.22	-6	-10	54
	877	CL cerebellum	6.47	28	-42	-30
	167	CL cerebellum	6.09	26	-48	-48
	27	CL cerebellum	5.42	2	-64	-32
	14	CL supplementary motor area	5.41	8	10	46
	95	IL cerebellum	5.39	-30	-48	-28

All clusters were significant at $p<0.05$ with familywise error correction. Cluster volume=number of 8 mm^3 voxels in cluster; Peak T=peak t value within the cluster; IL=ipsilesional; CL=contralesional. For larger clusters, the locations of several local maxima within the clusters are listed.
